# Explanations of a magic trick across the life span

**DOI:** 10.3389/fpsyg.2015.00219

**Published:** 2015-03-06

**Authors:** Jay A. Olson, Irina Demacheva, Amir Raz

**Affiliations:** Cognitive Neuroscience Laboratory, Psychiatry Department, McGill UniversityMontreal, QC, Canada

**Keywords:** magic, magical beliefs, magical thinking, appearance–reality distinction, conjuring

## Abstract

Studying how children and adults explain magic tricks can reveal developmental differences in cognition. We showed 167 children (aged 4–13 years) a video of a magician making a pen vanish and asked them to explain the trick. Although most tried to explain the secret, none of them correctly identified it. The younger children provided more supernatural interpretations and more often took the magician's actions at face value. Combined with a similar study of adults (*N* = 1008), we found that both young children and older adults were particularly overconfident in their explanations of the trick. Our methodology demonstrates the feasibility of using magic to study cognitive development across the life span.

## 1. Introduction

Magic tricks depend on assumptions about the world. Magicians skillfully violate these assumptions to create mysteries (Rensink and Kuhn, 2014). Since assumptions change with age, magicians perform differently for children and adults. Children, for example, may prefer watching physical magic such as vanishing objects, while adults can understand psychological magic such as mind-reading. To keep performances suitable, magicians have developed intuitions about which tricks work best for which ages (e.g., Ginn, 2004; Kaye, 2005). Examining these intuitions could lead to new insights or methods in the study of cognitive development.

Scientists have leveraged magic to explore other areas in psychology (Kuhn et al., 2008) including attention, perception, decision-making, and problem solving. Some have used both children and adults in their samples to compare cognitive development (e.g., Subbotsky, 2001). Few, however, have explored developmental differences with a large sample over a wide age span. Combined with previous research on adults (Demacheva et al., 2012), we present a feasibility study of 1175 participants aged 4–90 years.

Due to their level of maturation, children have different expectations and assumptions than adults; magicians thus cater to them with a specific set of effects (Sharpe, 1988; Rissanen et al., 2014). Around 4 years of age, children begin to understand that other people have distinct beliefs and intentions—that is, they begin to form a Theory of Mind (Apperly et al., 2009). Around the same time, the distinction between appearance and reality becomes clearer (Flavell, 2000). When executive attention develops around 3–7 years of age, logical thinking and sustained attention improve (Posner and Rothbart, 2007). With these developments, children are better able to make assumptions about what is going to happen and thus become more receptive to magic tricks.

*Magical beliefs*—such as beliefs about the existence of events which violate physical laws—also change with age (Subbotsky, 2014). Young children tend to believe in fantasy figures (such as fairies; Phelps and Woolley, 1994; Woolley, 1997) and many preschool children believe magicians have supernatural powers (Evans et al., 2002). During school age, children start to develop a more scientific perspective which can override magical beliefs (Subbotsky, 2010). Even so, these beliefs can persist into adulthood. In one study, more than half of college students ascribed psychic abilities to someone performing tricks resembling clairvoyance and psychokinesis, even if he was introduced as an amateur conjurer (Benassi et al., 1980). In another study, adults who claimed not to believe in supernatural abilities were reluctant to let the experimenter cast a spell on their identification cards (Subbotsky, 2001). Though some magical beliefs decrease with age, they continue to play an important role throughout the life span (Subbotsky, 2014).

In this paper we present a preliminary study of magical beliefs in children and adults. Participants watched a magician make a pen vanish then they tried to explain the trick. This “non-permanence magic” (Subbotsky, 2001) surprises most people over 4 years old (Rosengren and Rosengren, 2007). We had three hypotheses:

1. Confidence in one's explanation of the secret will decrease with age. This is consistent with magicians' observations and with studies showing that young children feel overconfident in their cognitive abilities (Shin et al., 2007; Lipko et al., 2009).
2. Younger children (aged 4–8 years), compared to older ones, will show more magical beliefs when explaining the trick (see Phelps and Woolley, 1994).
3. Younger children (aged 4–5 years) will more often take observed events at face value, since the appearance–reality distinction is still developing (Flavell, 2000). Specifically, they will more often believe that the pen broke or dissolved in the magician's hands.

## 2. Methods

The experimenter led participants to a testing room with individual computers. The participants watched a recorded magic trick, tried to explain it, then rated their confidence in the explanation. Next, the experimenter prodded for alternative explanations using a questionnaire. Finally, participants explained the trick again and re-rated their confidence level. The entire procedure took under 30 min for each participant.

### 2.1. Participants

We recruited 167 children from a summer camp in Montreal, Canada. They were 8.8 ± 2.3 years old (mean ± SD, range 4–13) and around half (54%) were male. Each age group had at least ten participants (Table 1). The procedure conformed to the guidelines of the Jewish General Hospital Research Ethics Committee and we obtained parental consent.

**Table 1:** **Sample sizes and gender proportions for each age group**.

**Age**	**4–5**	**6**	**7**	**8**	**9**	**10**	**11**	**12**	**13**	**14–17**	**18–19**	**20–29**	**30–39**	**40+**
N	10	20	31	22	17	23	16	18	10	37	225	655	62	29
% Male	10	35	55	86	35	65	56	72	40	46	25	30	55	52

*Participants aged 13 and under completed the child version of the questionnaire; the rest did the adult version (Demacheva et al., 2012)*.

Previously we recruited a sample of 1008 participants 22.3 ± 6.6 years old (14–90, 31% male; see Table 1) which we used as a comparison group (Demacheva et al., 2012). They completed an analogous questionnaire online.

### 2.2. Materials

#### 2.2.1. Magic trick

**Figure 1:**
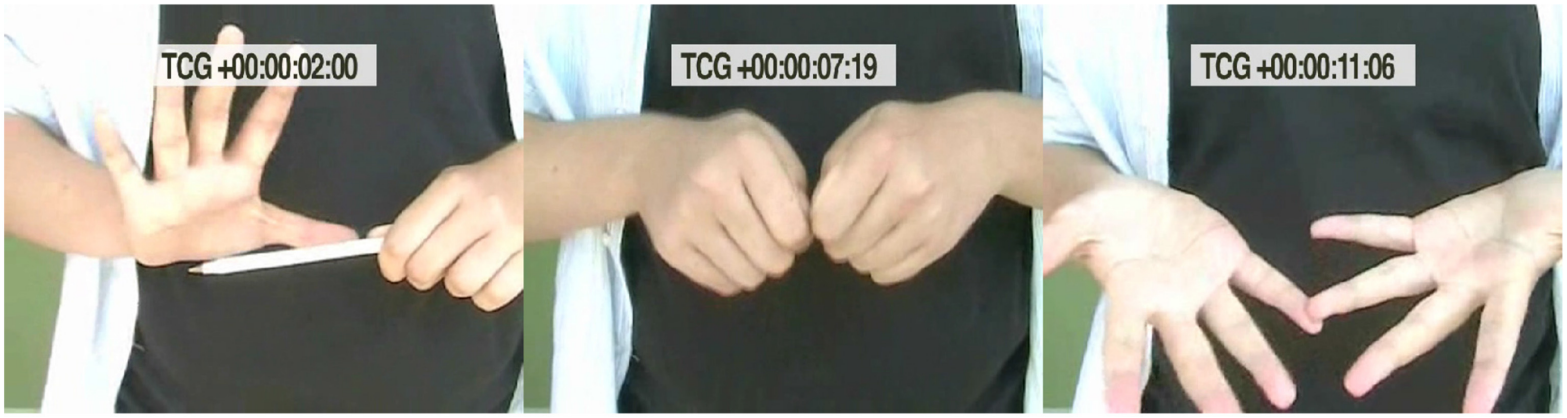
**Participants watched a silent video of a magician making a pen vanish**. For the video, see Supplementary Material.

The experimenter explained that we were studying how people think about magic tricks. On a computer, a 15-s silent video clip showed a magician making a pen vanish. In the video, the magician begins by showing a pen then appears to break it. When his hands open, the pen has vanished (Figure 1; see Supplementary Material for a video). We chose this minimal magic trick because it can fool both children and adults without needing patter, interaction, or explicit social cues (Demacheva et al., 2012; cf. Joosten et al., 2014). Participants could watch the video as many times as they wanted. Throughout the study, the experimenter referred to the magic trick in the video as a trick and avoided mentioning “real magic.”

There are several methods of performing this trick. Here, the secret involved the pen quickly moving inside the magician's jacket. A small cue in the video of an object hitting the magician's shirt hinted at this method. For a full description of the mechanism behind the trick, see Wilson (1988, p. 279, “The Vanish of the Handkerchief”).

#### 2.2.2. Questionnaire

The experimenter then led the children through a questionnaire (Appendix A in Supplementary Material); we used the same one as Demacheva et al. (2012) after a developmental psychologist adapted the wording for children. Most children tried to explain the secret of the trick. A magician who was unaware of our hypotheses later rated these explanations on a scale from 1 (i.e., completely wrong) to 5 (i.e., complete grasp of the method). Children rated their confidence in the explanations on a similar 5-point scale (1: not at all, 2: a bit, 3: some, 4: a lot, 5: a whole lot). The questionnaire then probed for alternative explanations by asking about required materials and possible methods to perform the trick. Some materials and methods were accurate (e.g., rubber bands, the pen moves quickly to a different location) and others were not (e.g., mirrors, the magician still holds the pen but it cannot be seen). Finally, children revised their initial explanations and re-rated their confidence.

## 3. Results and discussion

Consistent with our hypotheses, younger children gave more supernatural interpretations, more often took the magicians' actions at face value, and felt more confident in their explanations. Inconsistent with our hypotheses, confidence also increased with age among adults.

### 3.1. Secret

Although most children (62%, CI [54, 69%]^1^) tried to explain the secret, none correctly identified it. The magician gave 96% [92, 99%] of the initial explanations the lowest accuracy rating: completely inaccurate. (We considered the explanation correct if the magician rated it 3 or more out of 5). Even after being probed for alternative explanations, participants performed only marginally better: 2% [0, 6%] guessed it correctly. Adults similarly had little success in guessing the secret (5% were correct in their first explanation and 9% in the second; Demacheva et al., 2012). The trick was thus effective in that few people figured it out. We excluded these few from the rest of the analyses.

### 3.2. Explanations

**Figure 2:**
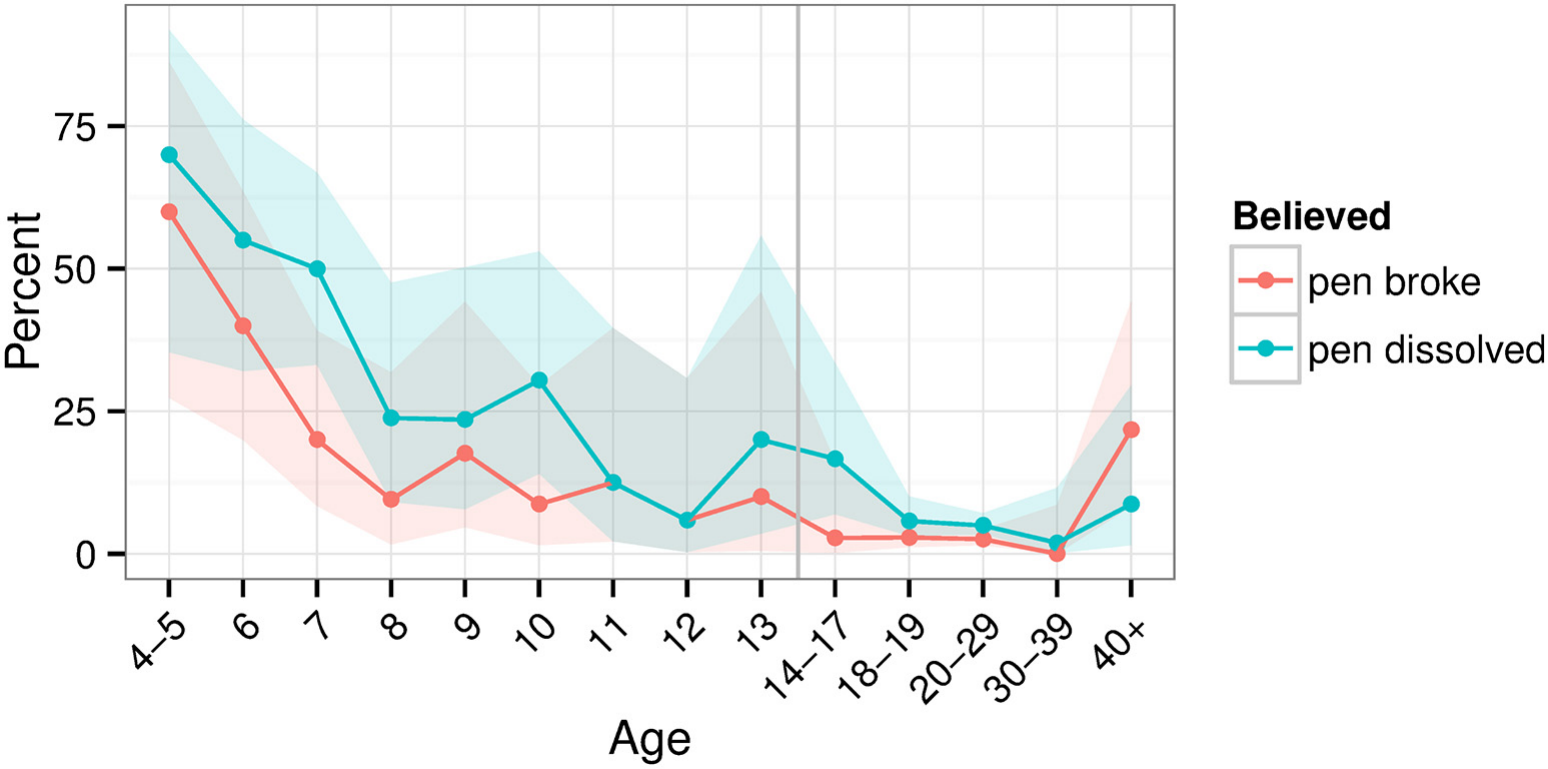
**Percent of participants believing that the pen broke or dissolved**. The vertical line separates those who took the child vs. adult version of the questionnaire. Shaded areas show bootstrapped 95% confidence intervals.

Attempts to explain the trick were broad. The 4–6-year-olds usually remarked the pen “just disappears” or the magician “just breaks it.” Indeed, the younger children more often took the magician's actions at face value. Specifically, they more often believed that the pen broke or dissolved in the magician's hands (Figure 2). Thus, age related to reports that the pen broke (χ^2^_(8)_ = 22.459, *p* = 0.004) or dissolved (χ^2^_(8)_ = 25.54, *p* = 0.001)^2^. These reports largely flattened out after the teenage years (Figure 2).

The 7–9-year-olds began to develop possible yet implausible explanations. Some suggested the magician hid the pen in his sleeves (which were rolled up in the video) or hid it in his skin. Others suggested the pen crumbled into smaller and smaller pieces until nothing remained. One suggested that the torso in the video was actually a mannequin and the magician hid the pen in the empty torso. The 10-year-olds and older children started to develop plausible explanations, such as a trick pen, camera tricks, or a hidden pocket. These progressive changes in the explanations presumably reflect both increased verbal ability and cognitive development.

**Figure 3:**
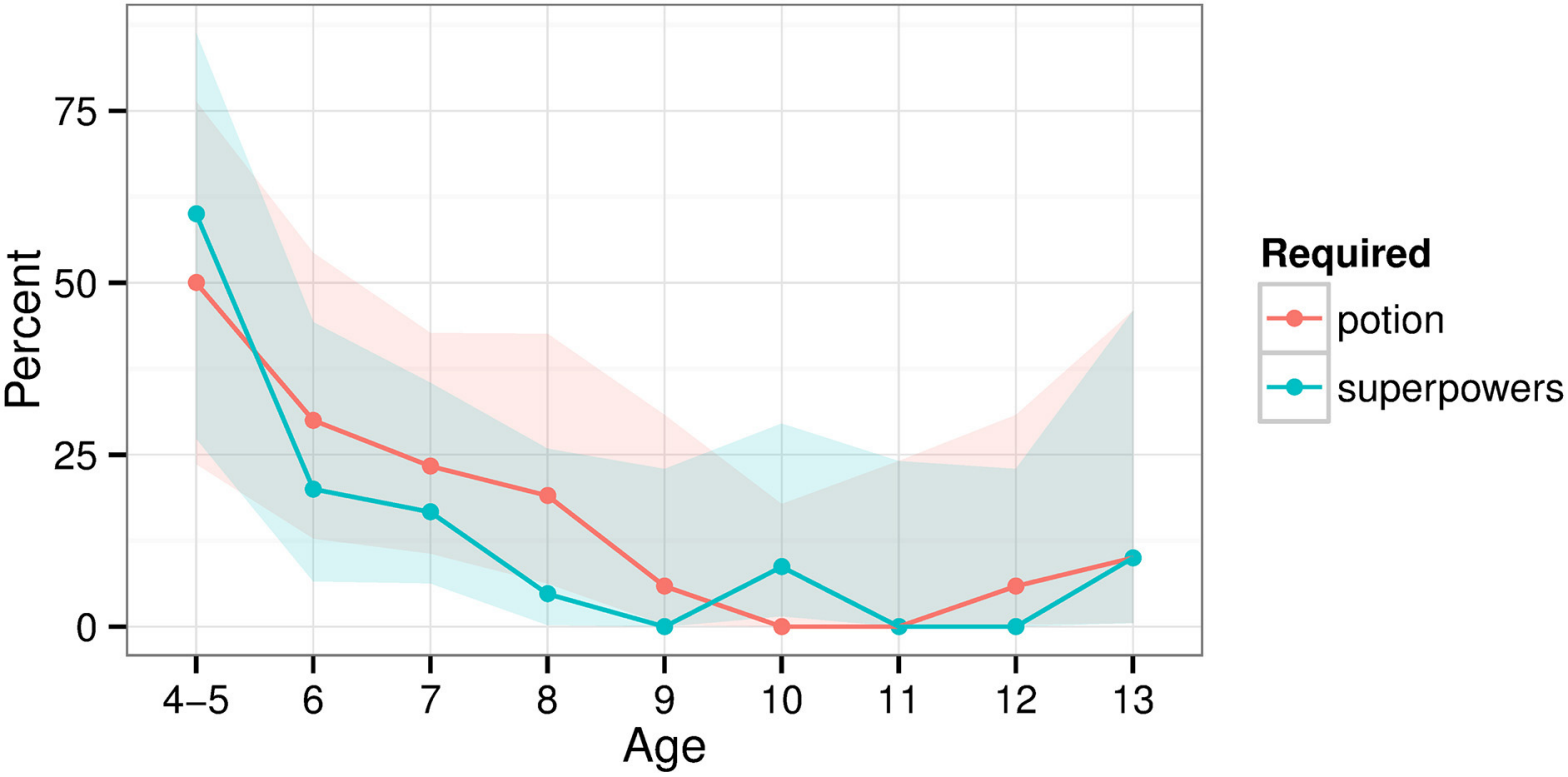
**Percent of participants believing that the magic trick required a magic potion or superpowers**. Shaded areas show bootstrapped 95% confidence intervals.

Consistent with previous studies (e.g., Evans et al., 2002), many of the younger children showed magical beliefs. Some suggested that the pen vanished simply because “the pen is magic.” When asked in the questionnaire, younger children more often believed the secret involved superpowers or a magic potion (e.g., “there is secret invisible stuff on his hands that makes [the pen] disappear”; Figure 3). There were thus relationships between age and the frequency of beliefs that the trick used a potion (χ^2^_(8)_ = 24.008, *p* = 0.002) or superpowers (χ^2^_(8)_ = 32.74, *p* < 0.001). The adult version of the questionnaire used different wording (“chemical reaction” rather than “magic potion”) which prevented a comparison to the children.

### 3.3. Confidence

Despite their lack of accuracy, children felt confident in their explanations: 84% reported at or above the midpoint of confidence. The majority (73%) reported “some” or “a lot” of confidence in their explanation. Adults reported roughly similar levels of confidence (57%).

**Figure 4:**
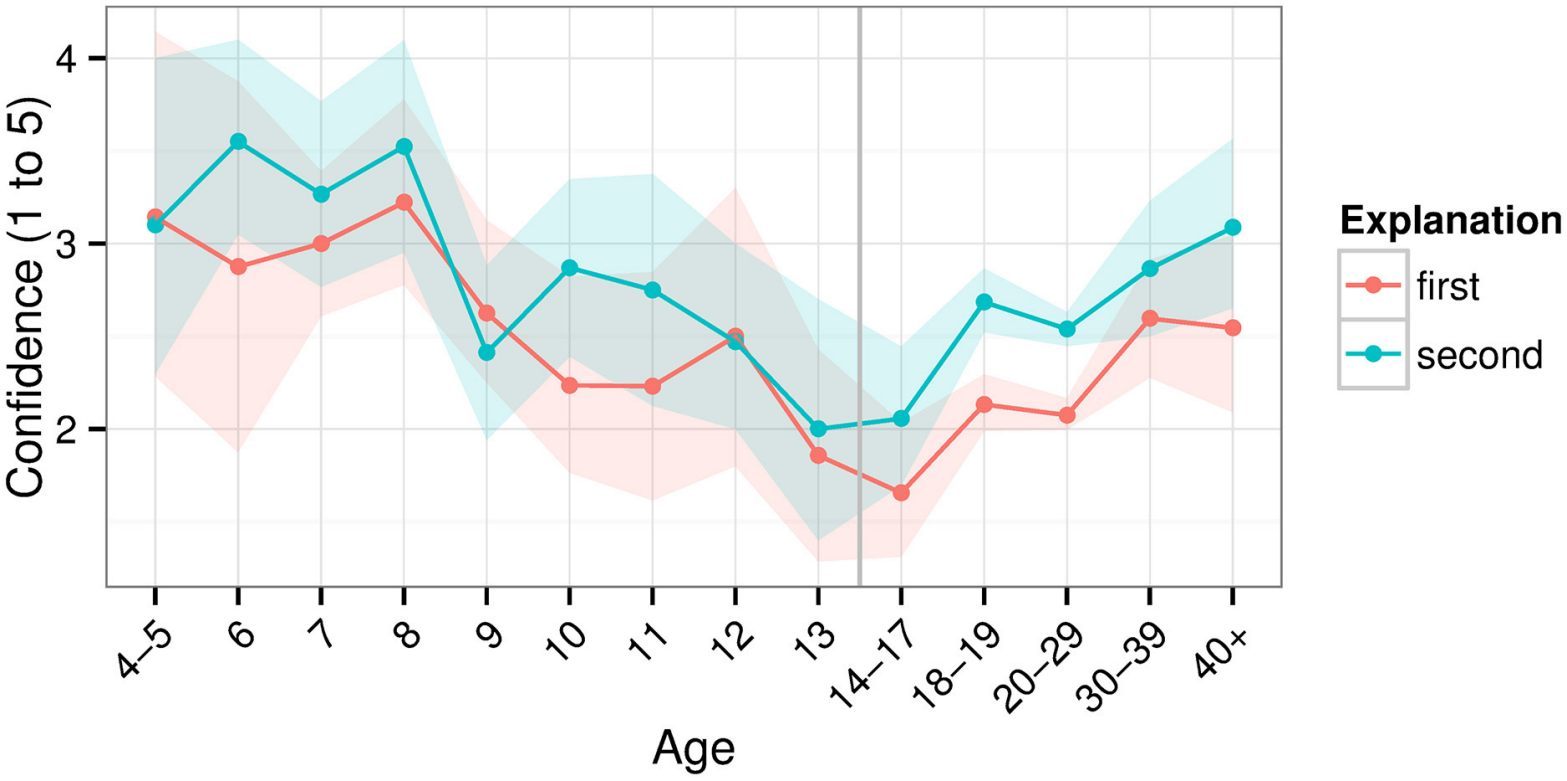
**Confidence in one's inaccurate explanation of the magic trick**. Shaded areas show bootstrapped 95% confidence intervals.

Among children, confidence seemed to decrease with age (Figure 4); there was a relationship between age and confidence in the explanation of the trick (first explanation: Kruskal-Wallis *H*_(8)_ = 15.509, *p* = 0.05; second: *H*_(8)_ = 19.176, *p* = 0.014). This general pattern is consistent with the finding that younger children are particularly overconfident (Lipko et al., 2009). Indeed, when presenting a deck of cards to young children, magicians (e.g., co-authors JO and AR) often hear, “Oh! I know that trick!.”

**Figure 5:**
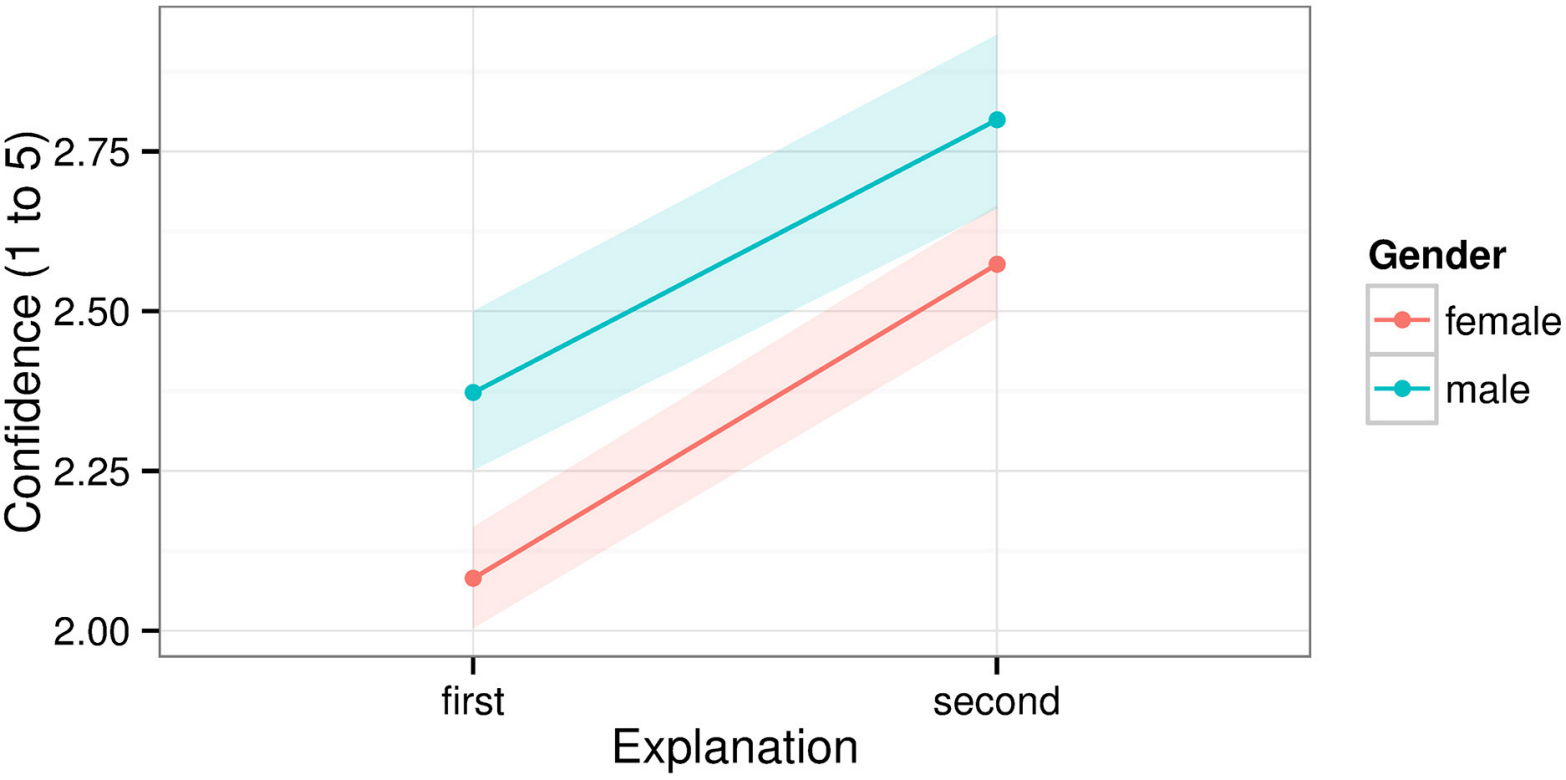
**Confidence in one's inaccurate explanation of the magic trick by gender**. Shaded areas show 95% bootstrapped confidence intervals.

Among adults, confidence seemed to *increase* with age (Figure 4). This seems inconsistent with findings that younger adults are generally more overconfident than older ones (Pliske and Mutter, 1996; Zell and Alicke, 2011). In our sample, gender differences may have contributed to this effect. Some studies have found that men are more overconfident in their abilities than women (Barber and Odean, 2001; Bengtsson et al., 2005). Our sample included more men as age increased above 18 (see Table 1) and overall males were more overconfident than females (Figure 5). The increase in males among older adults could have likewise increased confidence at older ages. Still, this could only explain part of the effect. Zell and Alicke (2011) found an interaction between age and overconfidence depending on which dimension was measured. For example, older adults were more confident about their sociability but less so about their athleticism. Perhaps, then, explaining magic tricks is a dimension showing more overconfidence with age. It remains unknown whether similar results apply to other magic tricks or cognitive tasks among adults.

### 3.4. Limitations

This study had three potential limitations. First, the questionnaires for children and adults differed slightly in wording (compare Appendix A in Supplementary Material here with Demacheva et al., 2012). Although we consulted a developmental psychologist to help ensure analogous wording, different results between children and adults could be partly due to inconsistencies in wording. To account for this, we minimized comparisons between those who took the child vs. adult version of the questionnaire. Second, the magic trick was recorded rather than performed live, which complicated the explanations of the trick. When young children claimed that the pen dissolved or vanished, they could have either intended that the pen actually vanished (in reality) or simply that it appeared to vanish (in the video); we could not differentiate these with certainty. Third, our methodology was insensitive to different interpretations of other questionnaire items. For example, when asked whether the trick needed “superpowers,” perhaps some children thought of supernatural abilities while others thought of specialized skills. One potential solution would be to perform the trick live each time followed by a more in-depth interview; in our case, this would have prevented such a large sample.

### 3.5. Implications

Using magic tricks may have several advantages for studying cognitive development across the life span. Traditional illusions in developmental psychology often require props such as boxes, screens, or backdrops (e.g., Baillargeon, 2002). These illusions can make the prop itself seem magical, such as when transforming objects inside a special box (e.g., Subbotsky, 2004). Using magic, as in the current study, the experimenter can make a person look magical rather than a prop. Shifting the locus of magic from props to people could help clarify differences in the development of magical beliefs regarding people vs. objects.

Further, unlike many of the illusions used to test phenomena like object permanence, magic tricks are robust across age: they amaze a large majority of people (here, 95%) over a wide age span. Many tricks work in diverse environments (e.g., Kuhn and Tatler, 2005) and can be translated for use in controlled experiments (Danek et al., 2014; Olson et al., 2015). Children and adults can thus view the same stimuli, which allows researchers to make more direct comparisons across different age groups. Such comparisons may be particularly useful to examine phenomena like magical beliefs or overconfidence which change their presentation across the life span (Benassi et al., 1980; Woolley, 1997; Zell and Alicke, 2011; Subbotsky, 2014). Similarly, magic tricks work across different cultures (Kiev and Frank, 1964) and thus could shed light on intercultural differences in magical beliefs.

In sum, our feasibility study demonstrated a method to test developmental hypotheses with large and diverse samples. Such a method combining video stimuli and online surveys is particularly useful to explore age-based changes in magical beliefs and overconfidence in children and adults. Magic may thus offer a useful tool to gain new insights in developmental psychology across the life span.

## Author contributions

JO wrote the manuscript and analyzed the data; ID designed the experiment, collected the data, and helped with the writing; AR helped with the design and manuscript revisions.

### Conflict of interest statement

The authors declare that the research was conducted in the absence of any commercial or financial relationships that could be construed as a potential conflict of interest.

## Footnotes

^1^Square brackets denote 95% confidence intervals (see Cumming, 2014).

^2^Statistical tests used data from participants 4–13 years old. Four and five-year-olds were combined due to their small sample sizes (see Table 1).

## References

[B1] Apperly I. A. Samson D. Humphreys G. W. (2009). Studies of adults can inform accounts of theory of mind development. Dev. Psychol. 45, 190–201. 10.1037/a0014098 19210001

[B4] Baillargeon R. (2002). The acquisition of physical knowledge in infancy: a summary in eight lessons, in Blackwell Handbook of Childhood Cognitive Development, ed Goswami U. (Malden, MA: Blackwell Publishers).

[B7] Barber B. M. Odean T. (2001). Boys will be boys: gender, overconfidence, and common stock investment. Q. J. Econ. 116, 261–292 10.1162/003355301556400

[B10] Benassi V. A. Singer B. Reynolds C. B. (1980). Occult belief: seeing is believing. J. Sci. Study Relig. 19, 337–349 10.2307/1386128

[B13] Bengtsson C. Persson M. Willenhag P. (2005). Gender and overconfidence. Econ. Lett. 86, 199–203 10.1016/j.econlet.2004.07.012

[B15] Cumming G. (2014). The new statistics: why and how. Psychol. Sci. 25, 7–29. 10.1177/0956797613504966 24220629

[B17] Danek A. H. Fraps T. von Müller A. Grothe B. Ollinger M. (2014). Working wonders? Investigating insight with magic tricks. Cognition 130, 174–185. 10.1016/j.cognition.2013.11.003 24300080

[B20] Demacheva I. Ladouceur M. Steinberg E. Pogossova G. Raz A. (2012). The applied cognitive psychology of attention: a step closer to understanding magic tricks. Appl. Cognit. Psychol. 1, 541–549 10.1002/acp.2825

[B24] Evans D. W. Milanak M. E. Medeiros B. Ross J. L. (2002). Magical beliefs and rituals in young children. Child Psychiatry Hum. Dev. 33, 43–58. 10.1023/A:1016516205827 12201181

[B27] Flavell J. H. (2000). Development of children's knowledge about the mental world. Int. J. Behav. Dev. 24, 15–23 10.1080/016502500383421

[B29] Ginn D. (2004). Professional Magic for Children. Loganville, GA: Scarlet Green.

[B31] Joosten A. Girdler S. Albrecht M. A. Horlin C. Falkmer M. Leung D.. (2014). Gaze and visual search strategies of children with Asperger syndrome/high functioning autism viewing a magic trick. Dev. Neurorehabil. 1–8. [Epub ahead of print]. 10.3109/17518423.2014.913081 24866104

[B35] Kaye D. (2005). Seriously Silly: How to Entertain Children with Magic and Comedy. Washington, DC: Kaufman & Company.

[B37] Kiev A. Frank J. D. (1964). Magic, Faith, and Healing: Studies in Primitive Psychiatry Today. Free Press of Glencoe.

[B39] Kuhn G. Amlani A. A. Rensink R. A. (2008). Towards a science of magic. Trends Cognit. Sci. 12, 349–354. 10.1016/j.tics.2008.05.008 18693130

[B41] Kuhn G. Tatler B. W. (2005). Magic and fixation: now you don't see it, now you do. Perception 34, 1155–1161. 10.1068/p3409bn1 16245492

[B43] Lipko A. Dunlosky J. Merriman W. (2009). Persistent overconfidence despite practice: the role of task experience in preschoolers' recall predictions. J. Exp. Child Psychol. 103, 152–166. 10.1016/j.jecp.2008.10.002 19058813

[B47] Olson J. A. Amlani A. A. Raz A. Rensink R. A. (2015). Influencing choice without awareness. Conscious. Cogn. [Epub ahead of print]. 10.1016/j.concog.2015.01.004 25666736

[B50] Phelps K. E. Woolley J. D. (1994). The form and function of young children's magical beliefs. Dev. Psychol. 30:385 10.1037/0012-1649.30.3.385

[B52] Pliske R. M. Mutter S. A. (1996). Age differences in the accuracy of confidence judgments. Exp. Aging Res. 22, 199–216. 10.1080/03610739608254007 8735153

[B54] Posner M. I. Rothbart M. K. (2007). Research on attention networks as a model for the integration of psychological science. Annu. Rev. Psychol. 58, 1–23. 10.1146/annurev.psych.58.110405.085516 17029565

[B57] Rensink R. A. Kuhn G. (2014). A framework for using magic to study the mind. Front. Psychol. 5:1508. 10.3389/fpsyg.2014.01508 25698983 PMC4313584

[B59] Rissanen O. Pitknen P. Juvonen A. Kuhn G. Hakkarainen K. (2014). Expertise among professional magicians: an interview study. Front. Psychol. 5:1484. 10.3389/fpsyg.2014.01484 25566156 PMC4274899

[B62] Rosengren K. S. Rosengren E. C. (2007). Discovering magic, in The Psychology of Harry Potter: An Unauthorized Examination of the Boy Who Lived, ed Mulholland N. (Dallas, TX: Ben Bella Books Inc.,)

[B65] Sharpe S. H. (1988). Conjurers' Psychological Secrets. Calgary, AB: Hades.

[B66] Shin H. E. Bjorklund D. F. Beck E. F. (2007). The adaptive nature of children's overestimation in a strategic memory task. Cogn. Dev. 22, 197–212 10.1016/j.cogdev.2006.10.001

[B69] Subbotsky E. (2001). Causal explanations of events by children and adults: can alternative causal modes coexist in one mind? Br. J. Dev. Psychol. 19, 23–45 10.1348/026151001165949

[B72] Subbotsky E. (2010). Magic and the Mind: Mechanisms, Functions, and Development of Magical Thinking and Behavior. New York, NY: Oxford University

[B74] Subbotsky E. (2004). Magical thinking in judgments of causation: can anomalous phenomena affect ontological causal beliefs in children and adults? Br. J. Dev. Psychol. 22, 123–152 10.1348/026151004772901140

[B77] Subbotsky E. (2014). The belief in magic in the age of science. SAGE Open 4, 1–17 10.1177/215824401452143325599003

[B83] Wilson M. A. (1988). Mark Wilson's Complete Course in Magic. Philadelphia, PA: Courage Books.

[B79] Woolley J. D. (1997). Thinking about fantasy: are children fundamentally different thinkers and believers from adults? Child Dev. 68, 991–1011. 10.2307/1132282 9418217

[B82] Zell E. Alicke M. D. (2011). Age and the better-than-average effect. J. Appl. Soc. Psychol. 41, 1175–1188 10.1111/j.1559-1816.2011.00752.x

