# Supplementary material for: Explanations of a magic trick across the life span
Source: Front Psychol. 2015 Mar 6;6:219. doi: 10.3389/fpsyg.2015.00219 (PMC4351568; doi:10.3389/fpsyg.2015.00219)
Supplement: Supplementary file 2 [file DataSheet1.PDF]

APPENDIX A: Questionnaire

The experimenter showed the children the magic trick then asked them the following questions. Common answers are shown in square brackets.

1. Have you seen this trick before? [88% no]
2. Do you think you know how magicians do this trick? [74% no]
3. Please tell us how magicians could do this trick. [62% offered an explanation other than “I don’t know”]
4. How confident are you of this explanation? (1: not at all, 2: a bit, 3: some, 4: a lot, 5: a whole lot) [$M = 2.64$]
5. Do you think this is a camera trick? [54% yes]
6. Please watch the video and write the specific TCG time [shown in the video] the pen leaves the magician’s hands. [$M = 6.92$ s; secret around 4–5 s, 17% answered within this range. Children had difficulty answering this question so it was kept only for consistency with the adult version of the questionnaire.]
7. What would you need to do this trick?[1]
   - String [14%]
   - *Safety pin(s)* [11%]
   - Magnets [19%]
   - Special lights [16%]
   - *Special clothing (for example: certain colour, with pockets)* [38%]
   - *Stickers* [7%]
   - *Rubber bands* [11%]
   - Magic potion [17%]
   - Mirrors [7%]
   - *Pen* [81%]
   - Other (specify) [1 answered “a hidden accomplice”]
8. How does the magician do it?[1]
   - He makes you look in the wrong spot. [19%]
   - He uses superpowers. [11%]
   - He still has the pen in his hands, but you cannot see it. [28%]
   - His magic potion eats up the pen. [15%]
   - He lets the pen fall. [35%]
   - He makes you forget what you saw. [14%]
   - *He quickly moves the pen from one location to another.* [43%]
   - Other (specify). [0%]
9. Please check what you think is **true**:[1]
   - *The magician is using a real pen.* [41%]
   - The pen actually breaks. [19%]

[1] None or multiple can be selected. Emphasis here shows correct (or plausible) items.

- The magician drops the pen. [34%]
- This special pen dissolves in magician's hands. [32%]
- The magician hides the pen between his fingers. [21%]
- None of the above. [13%]

10. How many times did you watch this video altogether? [$M = 5.42$]
11. Do you think that your first explanation for this trick is still good? [66% yes]
12. Please try to explain the trick in a better way.
13. How confident are you of this explanation? (1: not at all, 2: a bit, 3: some, 4: a lot, 5: a whole lot) [$M = 2.96$]
